# One Plus One is Better than Two: An Approach Towards a Single Blastocyst Transfer Policy for All IVF Patients

**DOI:** 10.1055/s-0042-1743096

**Published:** 2022-05-16

**Authors:** Pedro Felipe Magalhães Peregrino, Tatiana Carvalho de Souza Bonetti, Alecsandra Prado Gomes, Hamilton de Martin, José Maria Soares Júnior, Edmund Chada Baracat, Pedro Augusto Araújo Monteleone

**Affiliations:** 1 Centro de Reprodução Humana Monteleone, São Paulo, SP, Brazil; 2 Centro de Reprodução Humana “Governador Mario Covas”, Discipline of Gynecology, Department of Obstetrics and Gynecology, Hospital das Clínicas da Faculdade de Medicina, Universidade de São Paulo, São Paulo, SP, Brazil; 3 Molecular Gynecology Laboratory, Department of Gynecology, Escola Paulista de Medicina, Universidade Federal de São Paulo, São Paulo, SP, Brazil; 4 Centro de Neurociências e Saúde da Mulher “Professor Geraldo Rodrigues de Lima”, Neuroscience Discipline of the Department of Neurology and Neurosurgery, Escola Paulista de Medicina - Universidade Federal de São Paulo, São Paulo, SP, Brazil

**Keywords:** single embryo transfer, freeze-only, multiple pregnancy, in vitro fertilization, pregnancy rate, transferência de embrião único, criopreservação de todos os embriões, gestação múltipla, fertilização in vitro, taxas de gravidez

## Abstract

**Objective** It is known that the single embryo transfer (SET) is the best choice to reduce multiples and associated risks. The practice of cryopreserving all embryos for posterior transfer has been increasingly performed for in vitro fertilization (IVF) patients at the risk of ovarian hyperstimulation syndrome or preimplantation genetic testing for aneuploidy. However, its widespread practice is still controverse. The aim of this study was to evaluate how effective is the transfer of two sequential SET procedures compared with a double embryo transfer (DET) in freeze-only cycles.

**Methods** This retrospective study reviewed 5,156 IVF cycles performed between 2011 and 2019, and 506 cycles using own oocytes and freeze-only policy with subsequent elective frozen-thawed embryo transfers (eFET) were selected for this study. Cycles having elective SET (eSET, n = 209) comprised our study group and as control group we included cycles performed with elective DET (eDET, n = 291). In the eSET group, 57 couples who had failed in the 1 ^st^ eSET had a 2 ^nd^ eFET, and the estimated cumulative ongoing pregnancy rate was calculated and compared with eDET.

**Results** After the 1 ^st^ eFET, the ongoing pregnancy rates were similar between groups (eSET: 35.4% versus eDET: 38.5%; *p* = 0.497), but the estimated cumulative ongoing pregnancy rate after a 2 ^nd^ eFET in the eSET group (eSET + SET) was significantly higher (48.8%) than in the eDET group ( *p* < 0.001). Additionally, the eSET + SET group had a 2.7% rate of multiple gestations, which is significantly lower than the eDET group, with a 30.4% rate ( *p* < 0.001).

**Conclusion** Our study showed the association of freeze-only strategy with until up to two consecutive frozen-thawed eSETs resulted in higher success rates than a frozen-thawed DET, while drastically reducing the rate of multiple pregnancies.

## Resumo

**Objetivo** Sabe-se que a transferência de embrião único (SET) é a melhor escolha para reduzir as gestações múltiplas e riscos associados. A prática da criopreservação de todos os embriões para transferência posterior tem sido cada vez mais utilizada para fertilização in vitro (FIV), em especial quando há risco de síndrome de hiperestimulação ovariana ou realização de teste genético pré-implantacional. Entretanto, sua utilização disseminada ainda é controversa. O objetivo deste estudo foi avaliar a eficácia de duas SET sequenciais em comparação com uma transferência de embrião dupla (DET) em ciclos de FIV onde todos os embriões foram criopreservados.

**Métodos** Neste estudo retrospectivo foram revisados 5.156 ciclos de FIV realizados entre 2011 e 2019, e 506 ciclos usando oócitos próprios e criopreservação de todos os embriões com transferências eletivas subsequentes de embriões descongelados, foram selecionados para este estudo. Ciclos com transferência eletiva de embrião único (eSET, *n* = 209) compuseram nosso grupo de estudo e como grupo de controle incluímos os ciclos com transferência eletiva de dois embriões (eDET, *n* = 291). No grupo eSET, 57 casais que falharam na 1ª tentativa de eSET tiveram uma 2ª eFET e a taxa de gravidez em curso cumulativa foi estimada para o grupo eSET e comparada com o grupo eDET.

**Resultados** Após a 1ª eFET, as taxas de gravidez em curso foram semelhantes entre os grupos (eSET: 35,4% *versus* eDET: 38,5%; *p* = 0,497), mas a taxa de gravidez em curso cumulativa estimada após a 2ª eFET no grupo eSET (eSET + SET) foi significativamente maior (48,8%) do que no grupo eDET ( *p* < 0,001). Além disso, as taxas de gestação múltipla foram expressivamente inferiores no grupo eSET + SET (2,7%) quando comparado ao grupo eDET (30,4%; *p* < 0,001).

**Conclusão** Nosso estudo mostrou que a associação das estratégias de congelamento de todos os embriões com até duas eSETs sequenciais resultou em maiores taxas de sucesso do que uma DET com embriões descongelados, além de reduzir drasticamente a ocorrência de gestações múltiplas.

## Introduction

The ultimate goal of assisted reproduction techniques (ART) is to offer patients the highest chance of having a healthy live birth. However, multiple pregnancies, which carry an increased risk of complications for both fetuses and mothers, is yet a very common condition in ART. Since the first successful conception via in vitro fertilization (IVF) 40 years ago, advances in protocols have resulted in increasing success rates. Nowadays, in the era of personalized medicine, the practice of fixed protocols is becoming outdated and defining individualized parameters for each situation is now considered more appropriate for obtaining higher success rates, that is to say ongoing pregnancy rates (PRs), and fewer adverse effects, such as multiple pregnancies, of IVF.

Among these advances, the improvement in embryo culture, better embryo selection techniques, and excellent results after embryos vitrification have allowed for better planning of cycles and embryo transfers, also allowing the transfer of a smaller number of embryos without impairment in the outcomes. The single embryo transfer (SET) is the ideal approach to reduce multiple pregnancies. 1 However, despite of the recommendations for a reduction in the number of embryos transferred, 2 the double embryo transfer (DET) is still the most common practice worldwide, with multiple pregnancies remaining the most important iatrogenic complication of ART. 3

Efforts to stimulate SET were made, mainly based on studies demonstrating the transfer of two embryos in sequential SET cycles, with results in similar cumulative live birth rates compared with DET, and reduced multiple pregnancy rates. 4 However, SET is preferentially practiced in the good prognosis couples 5 or associated with preimplantation genetic testing for aneuploidy, which also advocates the freeze-only strategy for most cases. The freeze-only strategy has been increasingly used, in which all available good-quality embryos are frozen, and transfers are delayed for a natural or hormone replacement cycle. This practice is supposed to allow for better synchrony between blastocyst and endometrium maturation, which could hypothetically improve the overall outcomes due to a temporal interaction between an implantation-competent blastocyst and a receptive endometrium. 6 7 The main indication of the freeze-only strategy is for patients at the risk of ovarian hyperstimulation syndrome (OHSS) 8 and those undergoing preimplantation genetic testing for aneuploidy. 9 Despite some authors, who defend the superiority of frozen-embryo transfers' regimen as compared with the fresh embryo transfer strategy in both normal and high-responders, 10 11 there is no consensus on the widespread use of the freeze-only strategy. 12

Based on the perception that the combination of freeze-only strategy and SET is an efficient approach, we aimed in this study to evaluate retrospectively the outcomes of the real routine IVF practice in a cohort of patients undergoing the freeze-only strategy, who underwent consecutive elective SET. As reference, we compared the outcomes with patients who had the same characteristics but underwent DET.

## Methods

### Study Design

This is a retrospective cohort study evaluating freeze-only cycles performed as part of the routine care in a single assisted reproductive center. Written informed consent was obtained from all patients before treatment, consenting to the treatment procedures and to the use of their data in scientific publications with no patient identification.

This study is based on databank of anonymized data and according to local legislation it was exempt from approval by the Institutional Review Board and specific Informed Consent. The database included all IVF cycles performed between 2011 and 2019 at Monteleone Assisted Reproduction Center, São Paulo, Brazil.

The inclusion criteria were cycles of patients in which all embryos were cryopreserved (freeze-only cycles) and no fresh embryo transfers were placed. From 5156 cycles performed in the period of study, we selected 2725 freeze-only cycles. From those, we excluded cycles using donated oocytes, testicle sperm, embryo biopsy and more than 2 embryos transferred in the subsequent frozen-thawed embryo transfers (FET). Missing data were not a reason for case exclusion and all cycle analyzed had all essential data (associated to inclusion or exclusion criteria) and most of the additional information.

**Fig. 1:**
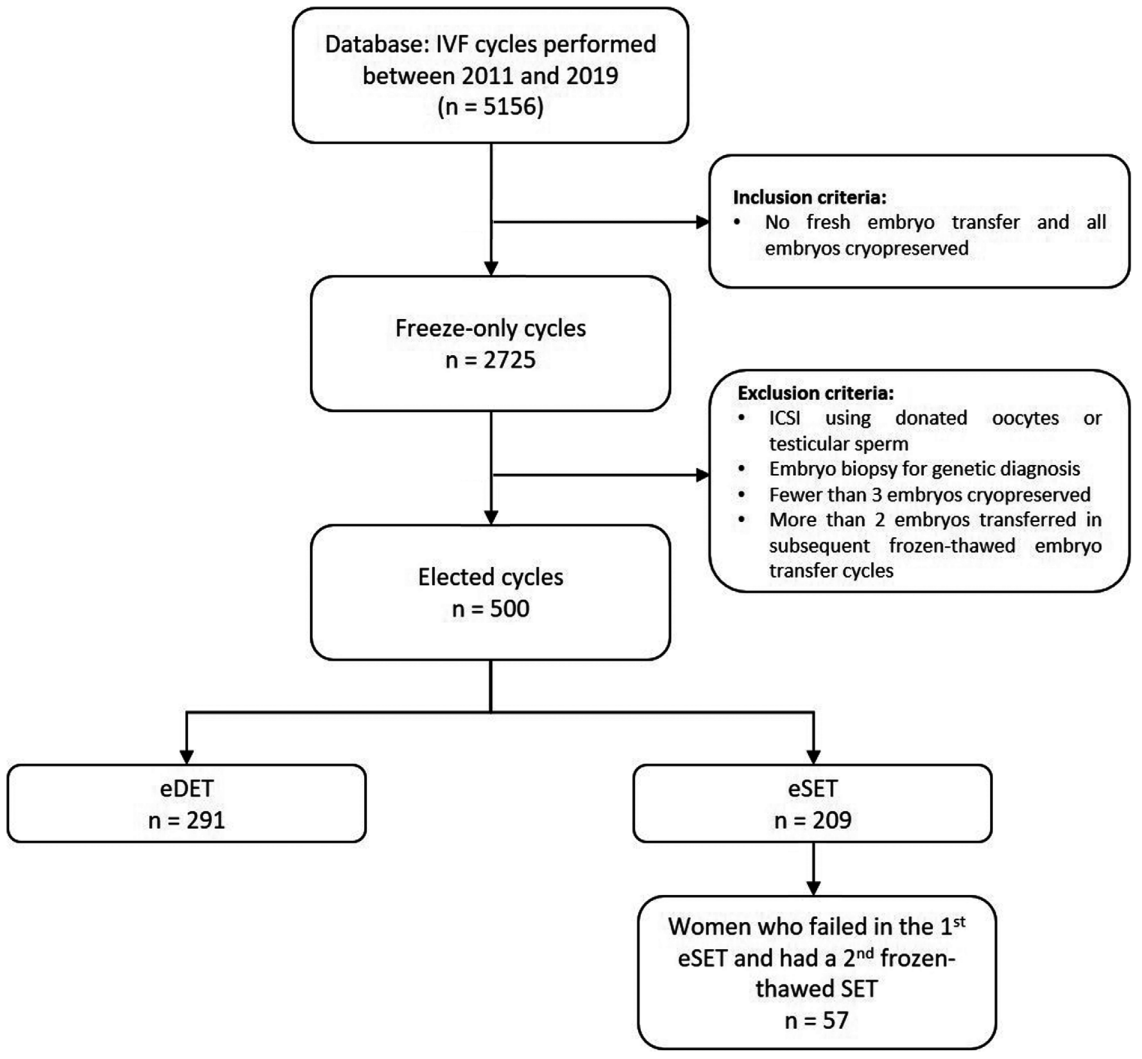
Workflow of study design.

The study group included 209 elective SET (eSET), in which patients underwent a SET in their first FET and had least one surplus embryo cryopreserved (eSET group). Among patients in the eSET group who did not become pregnant, 57 patients underwent a second frozen-thawed SET (eSET + SET). As a comparative group, we included 291 cycles in which two embryos were placed in the first FET and had least one surplus embryo cryopreserved composed the elective DET group (eDET group) ( Fig. 1 ).

### IVF Protocol

All patients underwent ovarian stimulation and oocyte pickup according to routine medical criteria. Briefly, pituitary blockage was performed with a GnRH antagonist (Cetrotide, Merck. Darmstadt, Germany). Ovarian stimulation was accomplished using recombinant Follicle-Stimulating Hormone (rFSH, Gonal-F, Merck. Darmstadt, Germany), with 150 IU/day as the starting dose for women up to 35 years of age and 225 IU/day for women older than 35 years. The dose was adjusted according to ovarian response. Follicular maturation was triggered when at least two follicles reached a diameter of 18 mm by using a GnRH agonist (Gonapeptyl, Ferring. Saint-Prex, Switzerland). Oocyte retrieval was performed after 35 to 36 hours by transvaginal ultrasound-guided aspiration. All oocytes were fertilized by Intracytoplasmic Sperm Injection (ICSI) 13 according to routine procedures; embryos were cultured using standard methods in a triple gas incubator (90% N2, 5% O2 and 6% CO2) at 37°C until vitrification.

All good quality embryos were vitrified on day 3 (D3) or day 5 (D5) using the Vitrification Freeze kit (Irvine Scientific. Santa Ana, CA, USA) with a Cryotip device (Irvine Scientific. Santa Ana, CA, USA), following the manufacturer's instructions. For warming, a Vitrification Thaw kit (Irvine Scientific. Santa Ana, CA, USA) was used. Embryos were evaluated by morphological criteria on D3 and/or D5. The embryos on D3 were considered good quality when they presented 8 to 10 symmetric blastomeres, with no multinucleation, and a maximum fragmentation level of 20%. 14 Blastocysts on D5 were considered good quality when they were expanded, with inner cell mass grade 3 or 4, and the trophectoderm was classified as A or B. 15

For FET, endometrial preparation was conducted with 100 μg of oestradiol valerate (Estradot, Novartis. Basel, Switzerland) for 14 days plus 800 mg of vaginal micronized progesterone (Utrogestan, Farmoquimica, FQM. Rio de Janeiro, RJ, Brazil) beginning 5 days before the transfer. Patients had standard endometrium evaluations through ultrasonography, and no other endometrial evaluations or procedures were performed. Embryos were thawed and evaluated for survival and morphology. For embryos cryopreserved at the cleavage stage, they were thawed, evaluated for survival, cultured until blastocyst stage, evaluated for morphology, and transferred. For embryos cryopreserved at blastocyst stage, they were warmed, evaluated for survival and morphology, and transferred in the same day. A higher quality blastocyst was always preferentially transferred.

### Data Collection and Statistical Analysis

The data were obtained from the clinical report forms and tabulated for this study. The groups were determined according to the number of embryos transferred (SET or DET). However, the choice for the number of embryos to be transferred was not controlled, as it was a shared decision between the couple and an assistant doctor. The woman's age, number of previous failed cycles, infertility factor, embryos quality, and the couple's choices were the factors that guided the number of embryos to be transferred, as is routine in the clinical practice.

The primary endpoint was an ongoing pregnancy, defined by the presence of a gestational sac with a heartbeat at 2 weeks after biochemical confirmation of pregnancy with serum β-hCG measurement. The ongoing pregnancy rate (PR) was calculated as the number of patients presenting a gestational sac with a heartbeat divided by the number of patients with embryos transferred, after the first transfer. Additionally, for the calculation of the cumulative ongoing PR, considering the 2 ^nd^ SET for patients who did not become pregnant in the 1 ^st^ SET (eSET + SET), we used a formula previously described by Luke et al. (2015): cumulative ongoing PR was equal to [ongoing PR for the 1st SET + ongoing PR for the 2nd SET * (1 - ongoing PR for the 1st SET)]. This calculation assumes no contraindication during cycle 1 for continuing into cycle 2, and estimates an outcome assuming that all patients who failed in the first cycle had a second SET.

The secondary outcomes evaluated in this study were multiple pregnancy rate (n° of patients with multiple gestational sacs divided by the total n° of patients with gestational sac), the implantation rate (IR) (n° of gestational sacs divided by the n° of embryos transferred) and miscarriage rate (n° of miscarriages divided by the n° of patients with gestational sac). Data analysis was performed using SPSS V.21 (IBM SPSS Software, USA). Normality distribution tests were performed, and patient demographic data were evaluated using descriptive statistics, including the means and frequencies. As data were normally distributed, parametric tests were used to compare means (Student *t* -test) of continuous variables. The Pearson chi-squared test was used to compare frequencies as appropriated. We considered *p* -values ≤ 0.05 to be statistically significant.

The sample power calculation was performed by using a two-tailed, two proportions calculation using the number of samples included in the study and cumulative ongoing PRs for eDET and eSET + SET groups. For the significance level (α) of 5%, the sample power (β) was 0.60.

## Results

Most of the couples included in the study were undergoing their first or second IVF cycles (88.1%). The demographic characteristics of the patients/cycles included in the study are presented in Table 1 and compared using the Student *t* -test.

**Table 1:** Demographic characteristics of patients and cycles included in the study

n	eSET	eDET	*p*
	209	291	
Age (years)	35.4 ± 3.9	34.9 ± 4.2	0.155
Body mass index (kg/m2)	22.5 ± 2.9	23.1 ± 3.6	0.092
Infertility time (years)	2.5 ± 1.7	2.9 ± 2.2	0.053
Basal FSH (IU/mL)	6.3 ± 5.0	6.3 ± 3.2	0.959
Total gonadotropin dose administered (IU)	1.809.4 ± 372.2	1.869.9 ± 481.4	0.129
Number of collected oocytes	20.0 ± 11.5	17.0 ± 9.2	0.002
Number of collected MII oocytes	15.2 ± 8.5	13.3 ± 7.4	0.008
Number of cryopreserved embryos	9.1 ± 4.5	8.8 ± 4.5	0.381

Note: According to the Student *t* -test. Abbreviations: eDET, elective double-embryo transfer; eSET, elective single-embryo transfer; FSH, follicle-stimulating hormone; MII, metaphase II.

For the first FET, we compared clinical outcomes between groups. The ongoing PRs and miscarriage rates were similar, while lower implantation and higher multiple PR rates were observed in the eDET group, as expected. The eSET group had two monozygotic twin pregnancies (2.7%), which is compatible with the incidence of twins in natural conception ( Table 2 ).

**Table 2:** Clinical outcomes of the study groups

	eSET	eDET	*p*
Number of transfers	209	291	
Implantation rate (%)	44.4%	29.7%	< 0.001
Ongoing pregnancy rate (%)	74/209 (35.4%)	112/291 (38.5%)	0.497
Miscarriage rate (%)	20/94 (21.3%)	26/138 (18.8%)	0.648
Multiple pregnancies rate (%)	2/74 (2.7%)	34/112 (30.4%)	< 0.001

Note: According to the continuity correction chi-square test. Abbreviations: eDET, elective double-embryo transfer; eSET - elective single-embryo transfer.

**Fig. 2:**
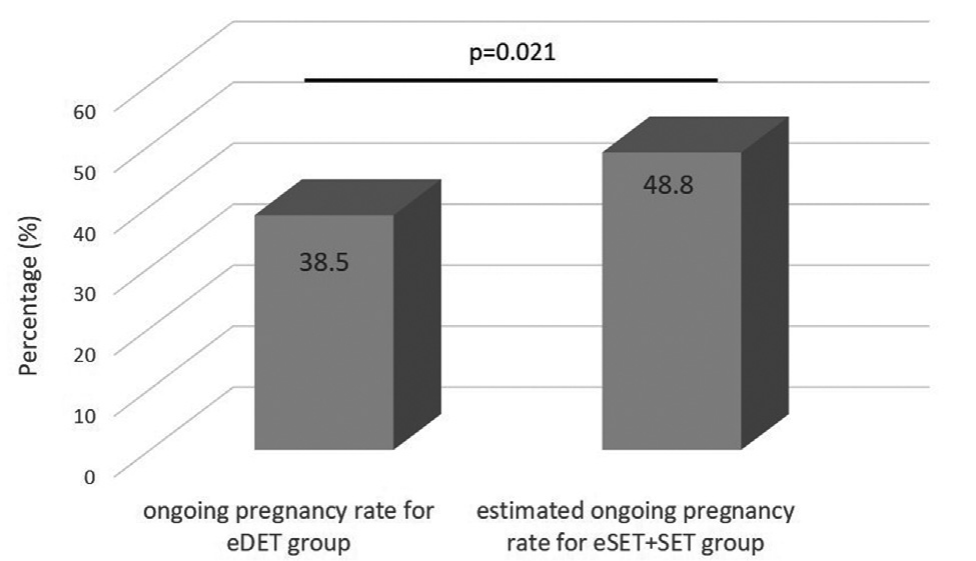
Cumulative clinical pregnancy rate after the transfer of two embryos in one transfer (eDET group) and in two single embryo transfers (eSET + SET group). Abbreviations: eDET, elective double-embryo transfer; PR, pregnancy rate; SET, single-embryo transfer.

In the eSET group, 135 women had their first transfer fail and 57 performed a second SET (eSET + SET, *n* = 57), resulting in 12 ongoing pregnancies (21.1%). Among the 78 remaining couples, 51 received two embryos in the second transfer with 18 ongoing pregnancies (35.3%). A total of 27 couples did not undergo a second transfer despite having embryos cryopreserved, resulting in a dropout rate of 20.0%. Our primary endpoint was to compare the clinical outcomes after the transfer of two embryos in one transfer cycle (eDET) or in two sequentially eSETs. The cycles in which an eSET failed in the first transfer, with none or a DET in the second transfer could not be included in the calculations. Then, we calculated the estimated cumulative ongoing PRs of the eSET + SET subgroup, according to the previously described formula, which assumes that all patients who failed in the first eSET would have received a second frozen-thawed SET. We named that result as estimated cumulative ongoing PRs for eSET + SET group. The comparison showed that the cumulative ongoing PR is significantly higher (10% higher) when two SETs are performed compared with the DET group ( Fig. 2 ).

## Discussion

Aiming to decrease the rate of multiple pregnancies and its consequences in IVF cycles, reproductive medicine and IVF societies stimulate the use of SET procedures. In general, the reduced number of embryos transferred for a maximum of two in most cases had a great impact for decreasing high order multiple pregnancies, but the rate of twin pregnancies is still excessive and heterogeneous globally. Despite several countries and clinics having introduced SET in their routine, there are several barriers that hinder the implementation of that practice. The female age is the foremost demographical factor influencing the number of embryos transferred, due to societal pressure on older women to have children, longer duration of infertility, and the reducing pregnancy chances with their own oocytes. The number of embryos available for transfer and their morphological quality is a biological factor that also influences this decision. In another facet of ART, three environmental aspects are important to mention; first, the financial issues are an important barrier, in which the insurance or government coverage of treatments, associated to the perception of transferring more embryos bringing a higher chance of pregnancy, can influence the choice of the number of embryos to be transferred. Societal, religious, and cultural factors can also impact the SET choice, as can some couples' desire to have twins regardless of the risks. Finally, the highly competitive market and the commercialization of ART services, drives the desire of providers to show higher success rates, even at the risk of high rates of multiple pregnancies. 16

On the other hand, the only efficient strategy to avoid multiple pregnancies in ART is SET. A randomized clinical trial (RCT) already showed the equivalence of two single embryo transfers compared with a double embryo transfer in different combinations almost 20 years ago. Thurin et al. 17 demonstrated the equivalence of one fresh SET plus one frozen-thawed SET compared with a fresh DET, with a dramatic reduction in multiple pregnancy rates. Following that, several studies demonstrated similar outcomes, 18 19 which in turn supports the recommendation of reproductive medicine societies to performing SETs.

Our study had a different outcome. We demonstrated that, besides the advantage of avoiding multiple gestation, two consecutive frozen-thawed SETs results in higher success rates than DET in one frozen-thawed cycle. The association of the freeze-only strategy with consecutive frozen-thawed SETs has not been extensively studied and most of the studies describe one SET in the fresh cycle and subsequent frozen-thawed ones. One retrospective study showed similar cumulative pregnancy and live birth rates after single and double frozen-thawed blastocyst transfers after a freeze-only strategy, 20 and another study evaluating the freeze-only strategy and SET in women with hypogonadotropic hypogonadism showed that SET is an effective strategy for decreasing the incidence of multiple conceptions while maintaining satisfactory live birth rates (50.5%). 21

Corroborating our hypothesis of better clinical outcomes after a freeze-only strategy plus SET, the American Society for Reproductive Medicine and the Society for Assisted Reproductive Technology (ASRM/SART) recommended SET for patients with a good prognosis aged < 38 years, except in cases in which the patient had several previous cycle failures and for whom DET was suggested. 5 Reinforcing the efficiency of the ASRM/SART recommendation, Eubanks et al. 22 evaluated retrospective data about clinical PRs before and after the ASRM/SART guidelines were published in 2017, as their clinic policy is to transfer no more than the recommended number of embryos. The study assessed patients < 38 years old using their own eggs and without preimplantation genetic testing, before (mean of 1.3 embryos transferred per patient) and after (all single embryo transfers) guideline revision. The outcomes showed that SET was very efficient in this population, as the overall live birth rates were maintained at around 50% after the reduction in the number of embryos transferred, and the twin PR rates decreased from 14.2% to 2.5%. 22

The differential characteristic of our study, compared with previous publications is that we used a freeze-only strategy, in which all embryos were cryopreserved by vitrification and transferred in subsequent hormone replacement cycles in the blastocyst stage, independently of the patients' age. Our population is composed of mainly good prognosis patients, considering a mean of 9 embryos cryopreserved per patient. But we showed better outcomes by using the sequential SETs approach and can discuss several hypotheses that support this unprecedented result. Some of the most important variables associated with the embryo implantation potential which have been the focus of several studies are: the embryo quality evaluated using conventional morphology, time-lapse morphokinetics, or preimplantation genetic test, 23 as well as the endometrium status, 24 and embryo-endometrial synchrony. 25

Studies have investigated progesterone action, endometrium gene, receptors, and protein expression based on data from the endometrial receptivity array. 26 27 More recently, the endometrium microbiome and its association with embryo implantation have been studied by the same research group. 28 Although the association of endometrium gene expression or microbiome with the implantation rates is controversial in the literature, 29 it is clear that there is a variability in the endometrial condition between cycles according to the patient's clinical condition or changes in the treatment approach. These variations in the endometrium performance can support the greater chance of implantation when performing consecutive SETs compared with one DET, as we transfer embryos in different endometrial conditions after one previous failure.

In the case of embryo feature, the vitrification technique is able to maintain the embryo implantation potential, 30 31 which, in turn, allows the performance of a freeze-only strategy and consecutive transfers in natural or hormone replacement cycles without impairment in the implantation potential. An extensive study evaluating more than 20,000 freeze-only cycles and FETs, in which all embryos transferred from that stimulation cycle were considered, demonstrated that the mean cumulative live birth rate was 50.0%, and dependent on the number of oocytes collected and the patient's age, recommending the applicability of the freeze-only strategy for the general population. 32 Besides that, the culture conditions, including time-lapse incubators and morphokinetic evaluation of embryos, preimplantation genetic test, and other add-ons can be applied to select embryos and encourage the practice of SET. 33

Considering the existence of variables that cannot be controlled in a clinical routine, and those inherent to the procedure that can be corrected in a subsequent cycle, it is reasonable to note that the transfer of embryos in separate events (consecutive SETs) will allow corrections, and produce a higher success rate than the transfer of two embryos in a single event. Our study confirmed the efficiency of the freeze-only strategy but, more importantly, we showed that consecutive SET is the better option for embryo transfer protocol and should be more extensively used. Owing to the retrospective nature of our study, we cannot exclude the possibility of residual interfering factors such as endometrium status or embryo ploidy, as these conditions were not evaluated in the cycles included in our study. A limitation of our study lies in the fact that not all patients who had a failed eSET in the 1 ^st^ transfer proceeded to a 2 ^nd^ SET cycle. Nevertheless, the calculation used in our study estimated the cumulative ongoing PRs as if all women who had a failed first SET had a second SET, confirming a clear advantage of SET over DET.

We must consider that the decision about the number of embryos to be transferred and their quality cannot be controlled, either. As a clinical routine, the number of embryos transferred is determined through a shared decision-making process between patients and doctors, after explaining the advantages and disadvantages of each procedure. Thus, the choice of the number of embryos to be transferred could have been influenced by the quality of the blastocysts available and the couple's preferences. Additionally, the percentage of couples undergoing their first or second cycle was a little higher in the eSET group (92.3%) than in the eDET group (85.1%), which can be another reason to choose DET for some couples. Although, it should be mentioned that our practice prioritizes transferring the best-quality frozen-thawed blastocysts available, all cycles included were elective (with at least one surplus frozen blastocyst), and the demographic characteristics of our study groups were similar, which made the comparisons possible.

Another point of our study that deserves attention is the similar ongoing pregnancy rates even after the first embryo transfer, while most studies show a higher pregnancy rate after DET when compared with SET. Most studies comparing the DET and SET strategies were performed in fresh cycles, which regards the condition of the endometrium after ovarian stimulation, or of FET after an unsuccessful fresh transfer, which means that the transferred embryo was not the first choice, and probably not the best quality one for that cycle. Our outcomes can be explained by the freeze-only strategy used in our study, and FET transferred the best quality embryo of the cohort in the hormone replacement cycle. Those outcomes can support the superiority of the freeze-all strategy even in cycles without classic indications, such as risk of OHSS or genetic evaluation of embryos. It is important to highlight that we excluded cycles with genetic analysis of embryos, and we did not have endometrium evaluations other than ultrasonography; therefore, we are studying only the regular embryo and endometrium evaluations.

## Conclusion

In summary, our study not only shows that eSET in freeze-only cycles maintains similar ongoing PRs to those of DET group after the first transfer, but also indicates that a second consecutive SET brings the best cost-benefit ratio, as it increases the success rates and decreases the rate of multiple pregnancies. Finally, several strategies can be used to avoid multiple pregnancies while keeping the satisfactory or desired success rates, and maybe it is time to consider that multiple embryo transfers should no longer be used in clinical practice. The freeze-only strategy and consecutive SETs can be an effective choice, as the one plus one approach is better than the two embryos transfer approach, leading to an increased chance of implantation and avoiding of multiple gestations.
